# Deciphering chicken gut microbial dynamics based on high-throughput 16S rRNA metagenomics analyses

**DOI:** 10.1186/s13099-015-0051-7

**Published:** 2015-02-26

**Authors:** Mohd Asrore Mohd Shaufi, Chin Chin Sieo, Chun Wie Chong, Han Ming Gan, Yin Wan Ho

**Affiliations:** Institute of Bioscience, Universiti Putra Malaysia, 43400, UPM, Serdang, Selangor Malaysia; Department of Microbiology, Faculty of Biotechnology and Biomolecular Sciences, Universiti Putra Malaysia, 43400, UPM, Serdang, Selangor Malaysia; Department of Life Sciences, International Medical University, Jalan Jalil Perkasa 19, Taman Esplanade, 57000 Kuala Lumpur, Malaysia; School of Science, Monash University Malaysia, Jalan Lagoon Selatan, 47500 Bandar Sunway, Selangor Malaysia

**Keywords:** Broiler chicken, Gut microbiota, Gastrointestinal tract, 16S rRNA, Metagenomics, Next-generation sequencing

## Abstract

**Background:**

Chicken gut microbiota has paramount roles in host performance, health and immunity. Understanding the topological difference in gut microbial community composition is crucial to provide knowledge on the functions of each members of microbiota to the physiological maintenance of the host. The gut microbiota profiling of the chicken was commonly performed previously using culture-dependent and early culture-independent methods which had limited coverage and accuracy. Advances in technology based on next-generation sequencing (NGS), offers unparalleled coverage and depth in determining microbial gut dynamics. Thus, the aim of this study was to investigate the ileal and caecal microbiota development as chicken aged, which is important for future effective gut modulation.

**Material and methods:**

Ileal and caecal contents of broiler chicken were extracted from 7, 14, 21 and 42-day old chicken. Genomic DNA was then extracted and amplified based on V3 hyper-variable region of 16S rRNA. Bioinformatics, ecological and statistical analyses such as Principal Coordinate Analysis (PCoA) was performed in mothur software and plotted using PRIMER 6. Additional analyses for predicted metagenomes were performed through PICRUSt and STAMP software package based on Greengenes databases.

**Results:**

A distinctive difference in bacterial communities was observed between ilea and caeca as the chicken aged (P < 0.001). The microbial communities in the caeca were more diverse in comparison to the ilea communities. The potentially pathogenic bacteria such as *Clostridium* were elevated as the chicken aged and the population of beneficial microbe such as *Lactobacillus* was low at all intervals. On the other hand, based on predicted metagenomes analysed, clear distinction in functions and roles of gut microbiota such as gene pathways related to nutrient absorption (e.g. sugar and amino acid metabolism), and bacterial proliferation and colonization (e.g. bacterial motility proteins, two-component system and bacterial secretion system) were observed between ilea and caeca, respectively (P < 0.05).

**Conclusions:**

The caeca microbial communities were more diverse in comparison to ilea. The main functional differences between the two sites were found to be related to nutrient absorption and bacterial colonization. Based on the composition of the microbial community, future gut modulation with beneficial bacteria such as probiotics may benefit the host.

**Electronic supplementary material:**

The online version of this article (doi:10.1186/s13099-015-0051-7) contains supplementary material, which is available to authorized users.

## Background

Microbial community in gastrointestinal tract (GIT) plays an important role in overall health and function of host, be it in human or animals. Numerous studies showed their contributions in many crucial roles such as in nutrient absorption, feed digestion and immune system [1-5]. Comprehensive analyses of the gut microbiota would lead to better understanding of the microbial interactions and biodiversity, which is important for implementing strategy to improve gut health.

Chicken gut microbiota has been studied previously using various approaches. The earliest reported was by using culture-dependent method [6,7]. This method can be bias and inaccurate as most bacteria are unable to be cultured due to unknown growth requirements [1,2,8-10]. Previous reports also highlighted that only up to 60% of caeca gut microbiota were culturable [6,7]. More advance techniques were introduced in the early 2000s, in which molecular fingerprinting methods such as denaturing gradient gel electrophoresis (DGGE) [11,12], temporal temperature gradient gel electrophoresis (TTGE) [8] and terminal-restriction fragment length polymorphism (T-RFLP) [1,2] were used. Sanger sequencing technology was also utilized by Lu *et al*. [13] to study the succession of chicken gut microbiota. Although these techniques were more robust than culture-dependent method, they were still incapable to represent the gut microbiota accurately due to its low coverage, throughput and semi-quantitative features [9,14,15]. In addition, these techniques were time consuming, costly and insufficient to reflect the true diversity of a diverse gut microbiota [10,14]. In recent years, the molecular technology is moving towards high-throughput next-generation sequencing (HT-NGS) which provides large scale analysis with unprecedented depths and coverages. Omics studies are possible with this kind of technology which enables a thorough and complex analysis of environmental communities [16]. Thus, the HT-NGS targeting on 16S rRNA genes was used in this study to investigate the diversity of chicken gut microbiota succession in ilea and caeca of broiler chicken fed with commercial feed. In this study, normal gut microbiota from ilea and caeca of chicken at age of 7, 14, 21 and 42 days were analysed. V3 region of 16S rRNA genes of samples were amplified and sequenced using HT Illumina NGS. Findings of this study provide fundamental knowledge on the gut microbiota composition of the chicken which can be contributed to the general well-being of the birds.

## Results

### Topographical and temporal differences in 16S sequences richness

**Figure 1:**
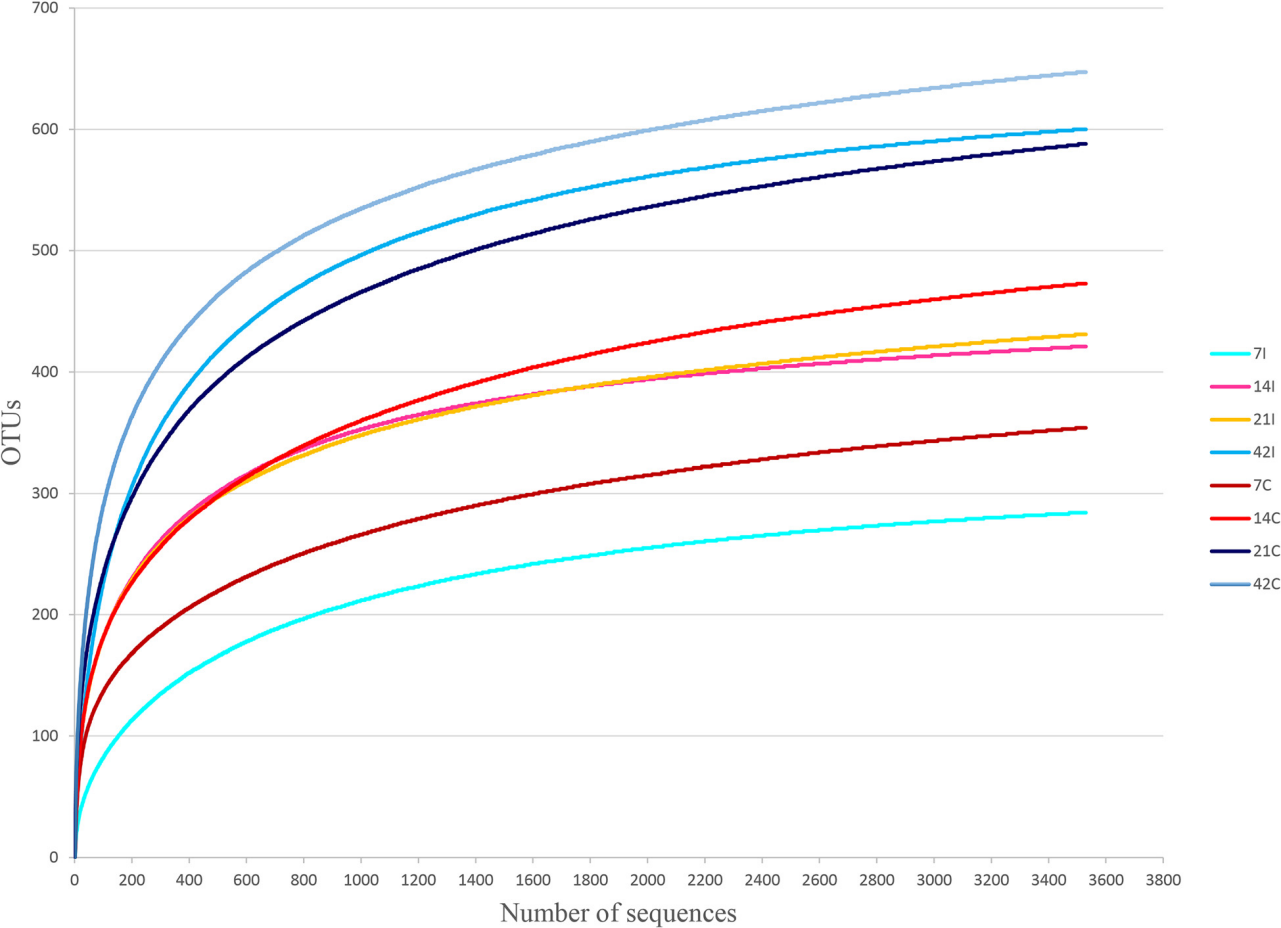
**Rarefaction curves of samples clustered at 95% sequences identity.** For each timepoint (Day 7, 14, 21 and 42) and part of intestine (I = ilea, C = caeca).

**Figure 2:**
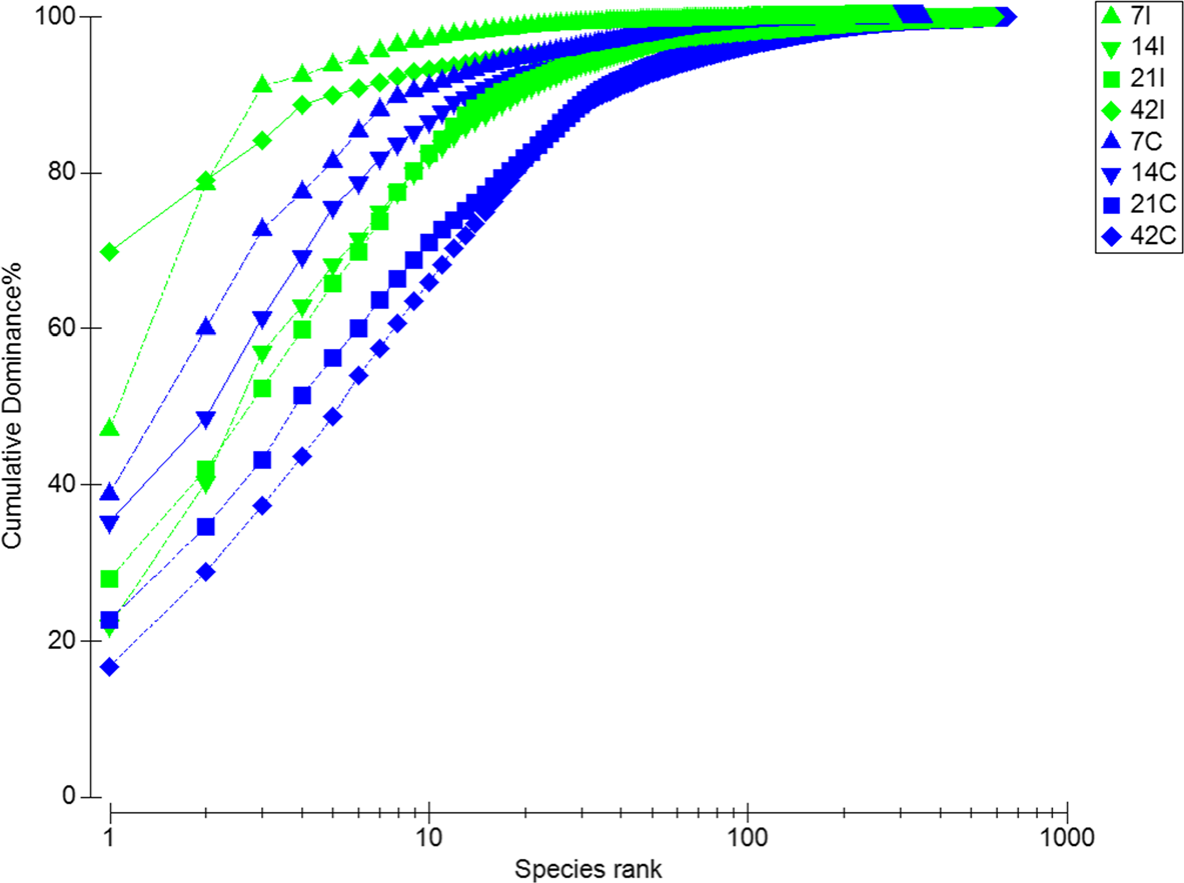
**K-dominance plot of cumulative percentage in relation to species rank.** For each timepoint (Day 7, 14, 21 and 42) and part of intestine (I = ilea, C = caeca).

A total of 3,456,387 sequence reads with a median length of 175 base pairs (bp) (V3 ~ 170–190 bp) were obtained from all samples. The sequences were further clustered into 3,694 operational taxonomic units (OTU) using a 95% similarity cut off. Rarefaction curves generated from the OTUs suggested that high sampling coverage (~99%) was achieved in all samples (Figure 1). Using ACE, Shannon and Inverse Simpson indices, a steady increase in species richness as the chicken aged was observed (Table 1). In addition, a higher bacterial diversity was obtained in caeca in comparison to ilea. Interestingly, elevated diversity coincided with a greater bacterial dominance in caeca over ilea (Figure 2). Nevertheless, both sites exhibited congruent increase in dominance over time.

**Table 1:** **OTUs (0.05% coverage) and diversity indices from samples at different timepoint and part of intestine**

**Samples**	**Number of observed OTUs**	**ACE**	**Shannon**	**Inverse Simpson**
7I	284	310.076	1.475	2.966
14I	430	455.014	3.312	12.869
21I	434	474.384	3.353	14.160
42I	603	630.024	3.347	11.936
7C	362	413.762	2.587	6.367
14C	487	552.406	3.663	17.758
21C	598	662.885	3.732	18.052
42C	660	706.335	4.365	34.077

For each timepoint (Day 7, 14, 21 and 42) and part of intestine (I = ilea, C = caeca). Total number of sequences normalized for each sample, n = 352,780).

### Bacterial taxonomic composition of ilea and caeca across time

**Figure 3:**
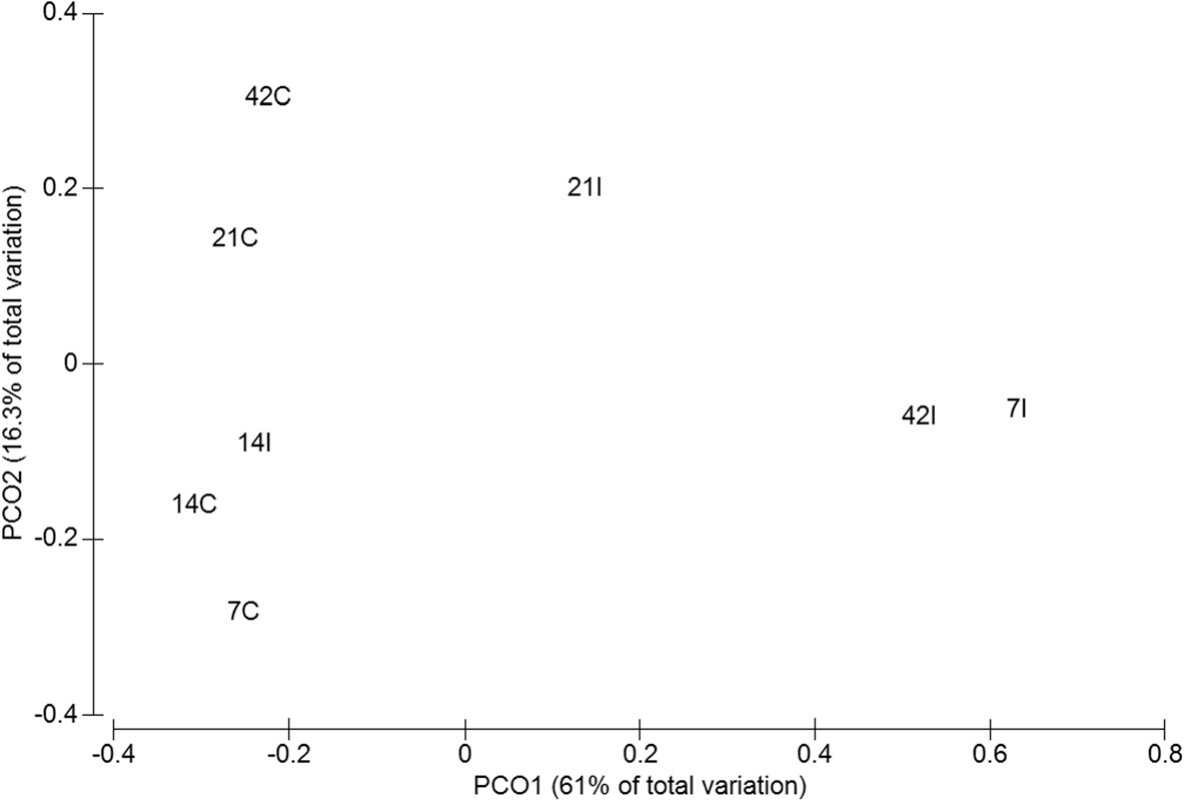
**Principal coordinate analysis (PCoA) of the community membership using Bray-Curtis distance.** For each timepoint (Day 7, 14, 21 and 42) and part of intestine (I = ilea, C = caeca).

Based on the ordination of the distance matrix generated using Bray-Curtis complementary algorithm, clear demarcation between bacterial assemblages from ilea and caeca were apparent along principal coordinate axis 1 (PCO1) of the PCoA plot (Figure 3). The separation was confirmed using analysis of molecular variance (AMOVA), P < 0.001) (Additional file 1: Table S1).

The compositional distribution pattern under different taxonomic classification including phylum, class, order, family and genus level were compared using PCoA. Three clusters separating phylum, order, class, family and genus was observed along axis PCO1 in the PcoA plots (Additional file 2).

**Figure 4:**
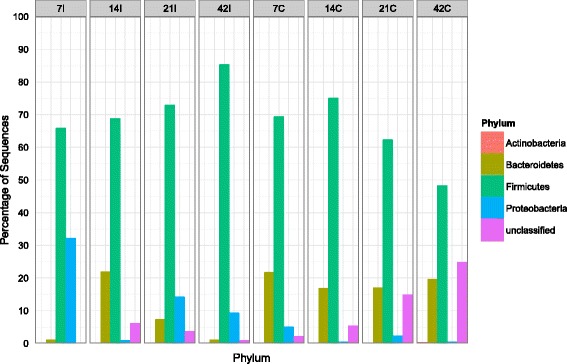
**Bacteria phyla distributions using V3 amplicon sequencing (n = Top 100 OTUs).** For each timepoint (Day 7, 14, 21 and 42) and part of intestine (I = ilea, C = caeca).

**Figure 5:**
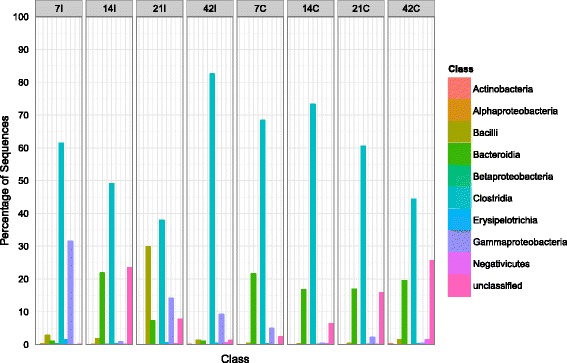
**Bacteria class distributions using V3 amplicon sequencing (n = Top 100 OTUs).** For each timepoint (Day 7, 14, 21 and 42) and part of intestine (I = ilea, C = caeca).

**Figure 6:**
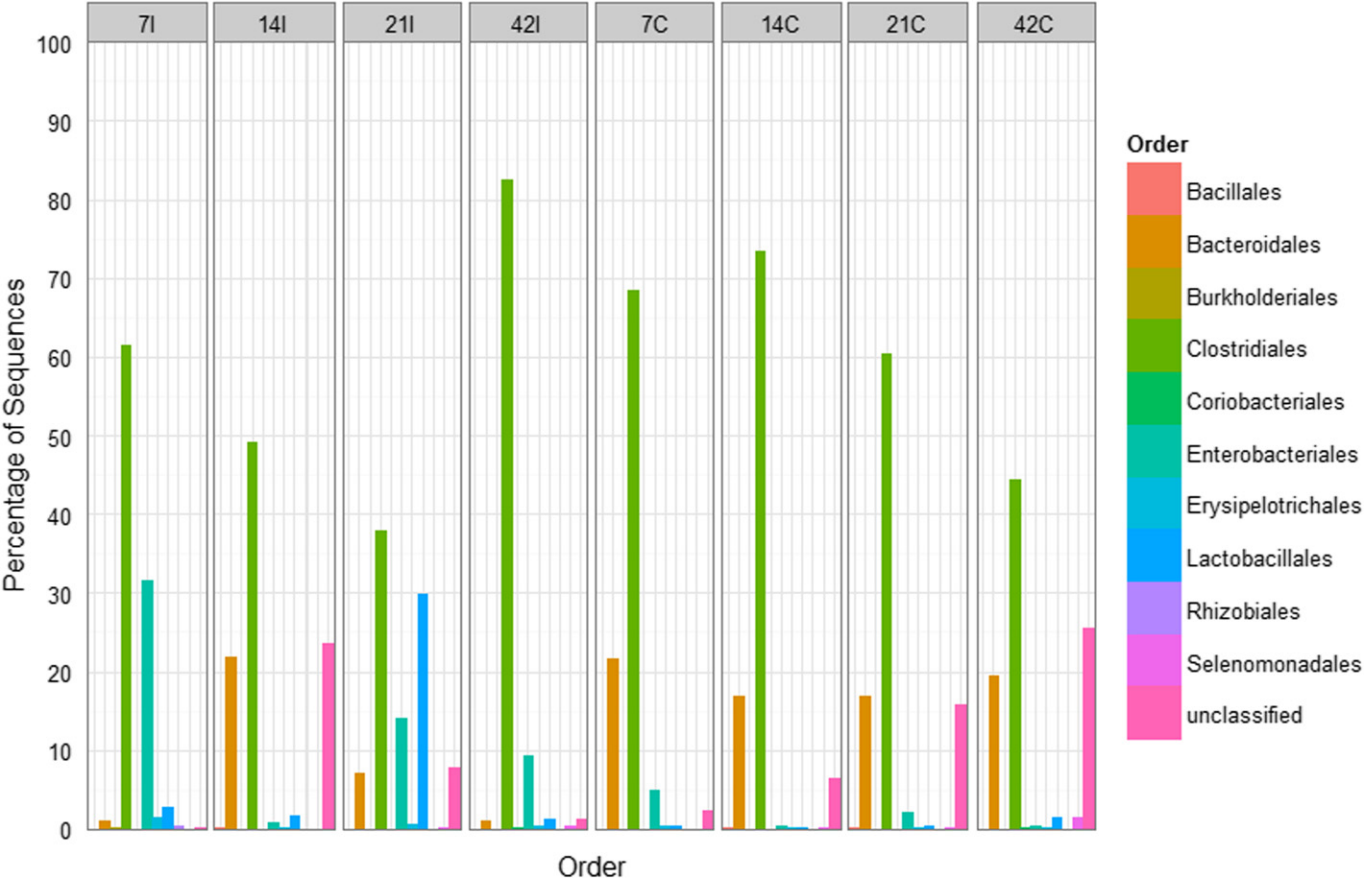
**Bacteria order distributions using V3 amplicon sequencing (n=Top 100 OTUs).** For each timepoint (Day 7, 14, 21 and 42) and part of intestine (I = ilea, C = caeca).

*Firmicutes* was the most abundant phylum (49-85%) in both ilea and caeca of chicken at all ages (Figure 4). Unlike in the caeca in which it increased from 69% at day 7 to 76% at day 14 and decrease to 49% at day 42, this phylum increased slowly in the ilea from 67% to 85% as the chicken aged. In ilea, *Proteobacteria* was the second most abundant phyla (5-32%), except at day 14 in which *Bacteroidetes* (22%) was more dominant. The presence of *Proteobacteria* was not obvious in caeca where it only can be detected at day 7 (5%) and 21 (3%). *Bacteroidetes* (18-21%) was consistently found as the second most abundant group at each time point in the caeca. From the phylum *Firmicutes, Clostridia* (38%-83%) was the most dominant class in the ilea and caeca of chicken at different age (Figure 5), with the members from order *Clostridiales* (Figure 6) being most abundant. In contrast to the caeca which exhibited a high proportion of *Bacteroidia* (17-22%; second most abundant class) (Order, *Bacterioidales*), the distribution of bacterial classes in the ilea was less consistent. On day 7, ilea were dominated by *Clostridia* (62%) and *Gammaproteobacteria* (32%) while on day 14, 21 and 42, the second most abundant classes shifted from *Bacteriodia* (23%) to *Bacilli* (30%) and *Gammaproteobacteria* (9%) respectively (Figure 5). *Enterobacteriales*, the most abundant order from the class *Gammaproteobacteria*, was higher in ilea (2-32%) compared to caeca (1-5%). *Lactobacillales*, a representative order from the class *Bacilli*, remained low (1-2%) in caeca samples although slightly higher population (2%) was observed at day 42 (Figure 6). Comparatively, higher population of *Lactobacillales* was observed in the ilea, approximately 1-3% in all samples except ilea of day 21, which recorded at 30%.

**Figure 7:**
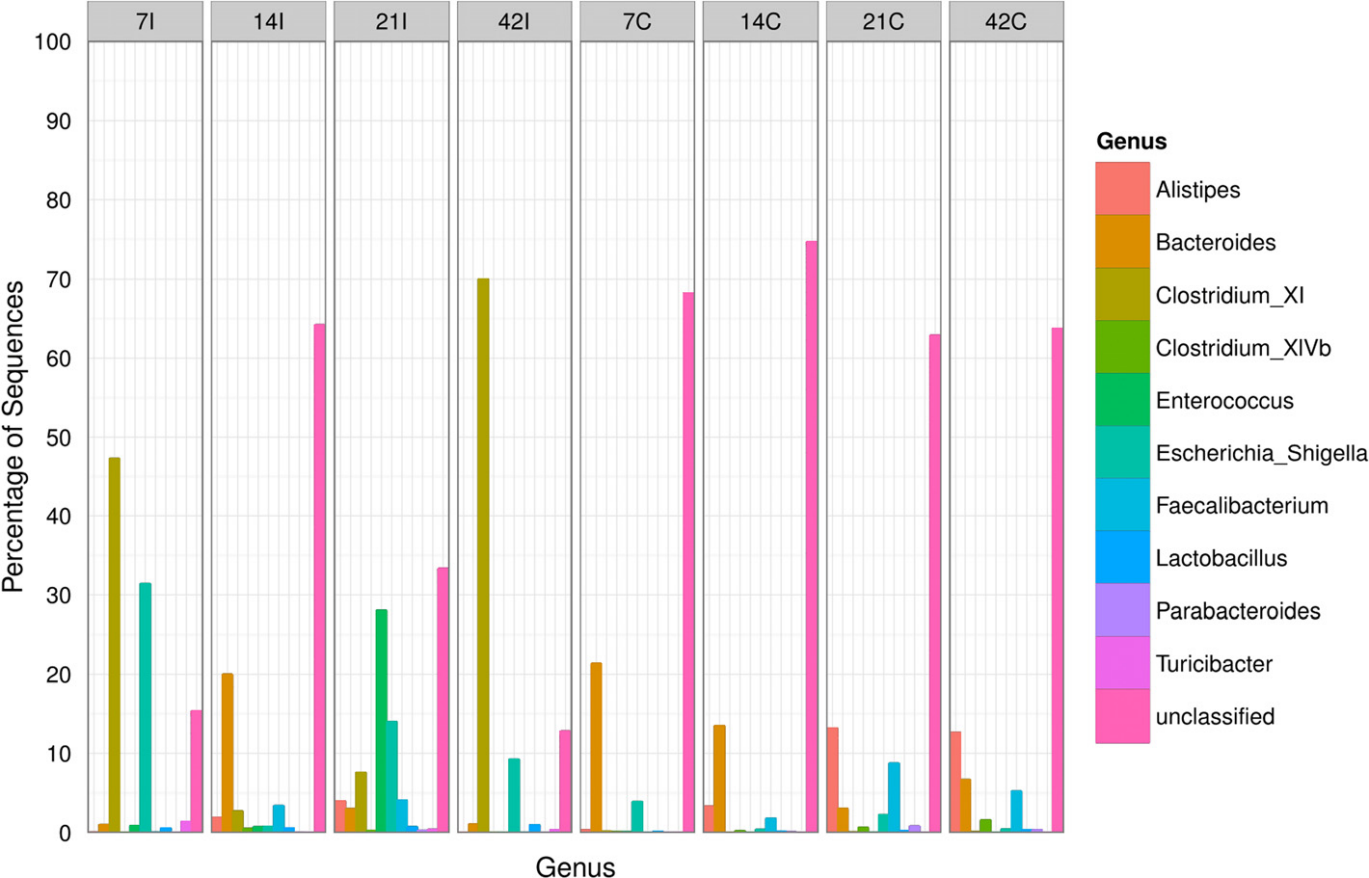
**Bacteria genera distributions using V3 amplicon sequencing (n = Top 50 OTUs).** For each timepoint (Day 7, 14, 21 and 42) and part of intestine (I = ilea, C = caeca).

At genus level, ilea was dominated by *Clostridium_XI* of *Clostridiaceae* family at day 7 (47%) and day 42 (70%) (Figure 7) During these two time points, high percentage of sequences for *Escherichia_Shigella* (Family, *Enterobacteriaceae*) (32% at day 7 and 9% at day 42) was observed. *Bacteroides* was found to be the major genera at day 14 (20%), followed by *Lactobacillus* (4%) and *Clostridium_XI* (3%). At day 21, the microbial diversity was more diversified in which it consisted of *Enterococcus* (28%), *Escherichia_Shigella* (14%), *Clostridium_XI* (7%), *Faecalibacterium* (5%), *Alistipes* (5%) and *Bacteroides* (4%). The microbial diversity in the caeca was less complicated and more consistent. It was mainly dominated by *Bacteroides* (3-22%), *Alistipes* (1-13%), *Faecalibacterium* (3-8%), *Clostridium_XIV* b (1-3%) and *Escherichia_Shigella* (1-5%).

### Predicted functional metagenomes in ilea and caeca

**Figure 8:**
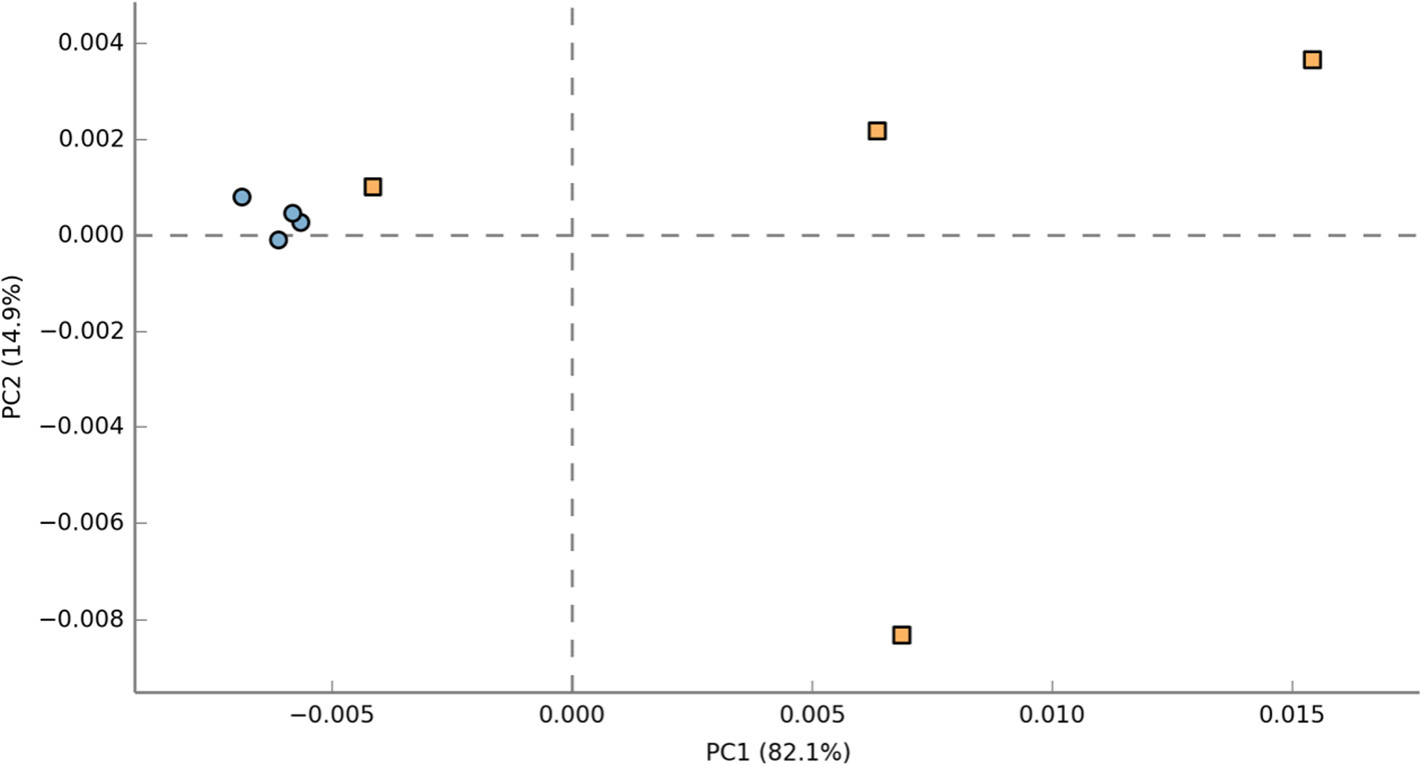
**Principal coordinate analysis (PCoA) of predicted functional metagenomes between ilea and caeca.** For part of intestine (Ilea = Orange, Caeca = Blue).

**Figure 9:**
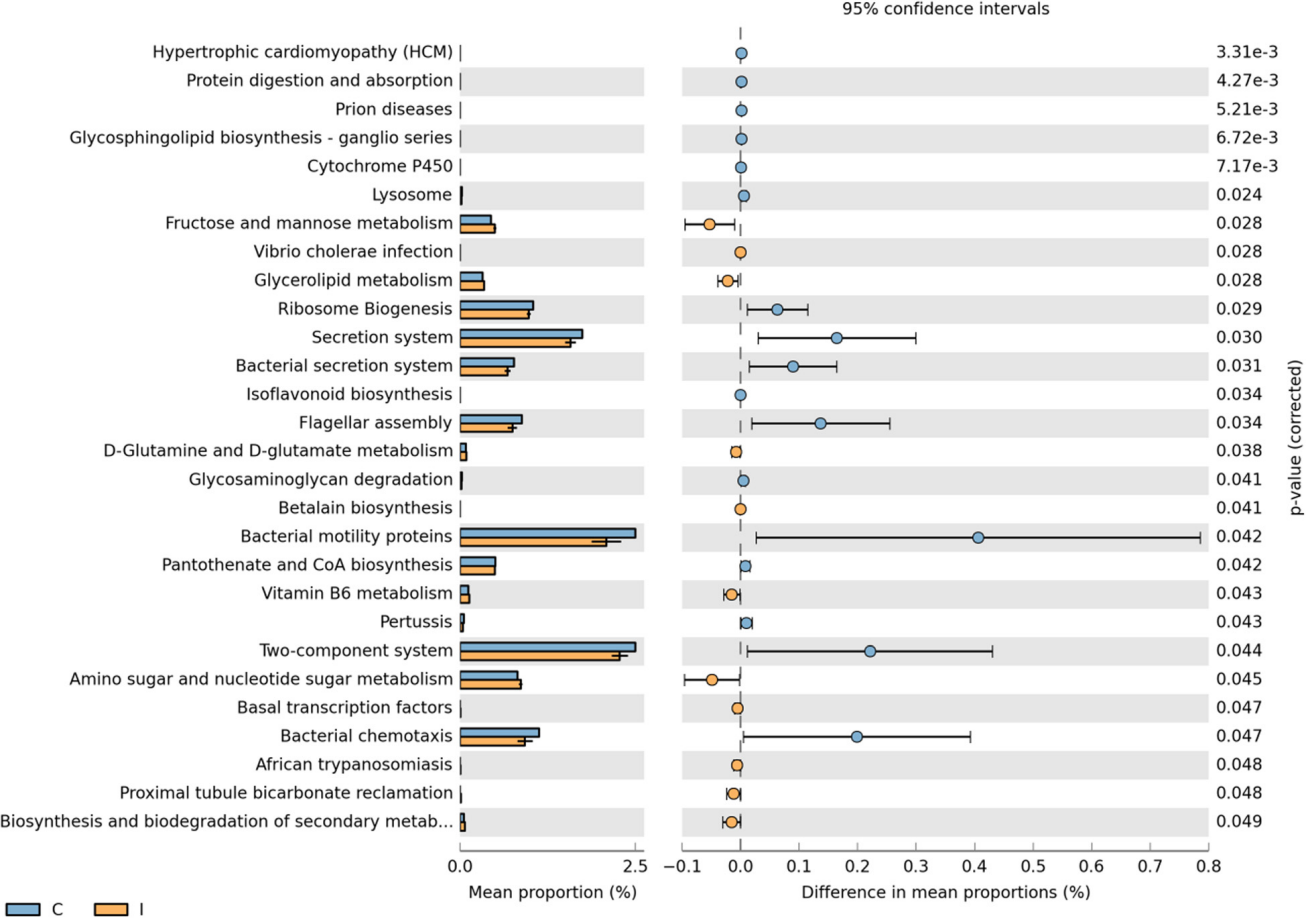
**Mean proportion and their differences in predicted functional metagenomes of the gut microbiota.** For part of intestine (Ilea = Orange, Caeca = Blue).

Based on the functionality prediction, a clear difference in the KEGG Orthologs (KO) composition between ilea and caeca was detected. The former formed a tight cluster on the left while the latter exhibited a sparse distribution along PC1 axis of the PCA plot (Figure 8). Among the 328 affiliated KEGG pathways, 28 was shown to achieve a statistical significant different at P < 0.05 (Figure 9). Notably, significant elevation in sugar and amino acid metabolism pathways was observed in ilea. On the other hand, pathways related to bacterial proliferation and colonization (e.g. bacterial motility proteins, two-component system and bacterial secretion system) was detected in caeca.

## Discussion

Thorough investigation of normal chicken gut microbiota is essential to understand their roles in host function. Nevertheless, available reports were mainly focusing on data obtained through the culture-dependent techniques [6,7] and early molecular fingerprinting methods [1,2,8,11-13,17]. Despite the extensive use of NGS in unravelling the function and importance of human gut microbiome [18-20], there is currently a lack of detail in biodiversity assessment using HT-NGS to understand the topological differences and development of gut microbiota in chicken intestines. Among the few available NGS based studies, Qu *et al.* [21] reported the effect of *Campylobacter jejuni* in chicken caeca, Danzeisen *et al.* [22] investigated the changes in chicken caeca microbiota based on anti-coccoidial and growth promoter treatments while Singh *et al.* [23] and Stanley *et al.* [4] performed metagenomics study of chicken faecal and caeca samples respectively, to investigate the difference between high and low feed conversion ratio (FCR) chicken. From previous studies, different regions of 16S rRNA were used for microbial classification. Danzeisen *et al.* [22] targeted on V3 region while Singh *et al.* [23] and Stanley *et al.* [4] focused on a combination of V1-V3 regions for sequencing. Studies integrating the V3-V4 region [24] and longer MiSeq read chemistry may provide a better resolution in microbial diversity and OTU classification [25], and thus, may resolve or validate some of the potential discrepancies between culture-based and culture-independent assessment of the chicken microbiota. Nevertheless, a single region such as V2 or V3 has also been reported to be able to discriminate microbial OTU up to genus level accurately [26]. Therefore, in this study, V3 region was used to investigate normal gut microflora of chicken intestines.

Based on our results, distinctive differences in gut microbiota richness and diversity between ilea and caeca were observed. This was shown based on the rarefaction curves (Figure 1) and diversity indices (Table 1) where caeca had significantly greater richness, diversity and variation in community structures than ilea. Ilea has been reported as a main site of nutrient absorption while caeca mostly as site of fermentation [5]. Caeca gut microbiota has crucial roles in breaking complex polysaccharides, uric acid, starch and cellulose [5,27]. According to the general consensus, gut microbial population becomes more complex as the chicken aged [11,12,22,28,29]. Our results were in agreement with this paradigm as a clear increasing trend was observed in the richness and diversity indices such as ACE, Shannon and Inverse Simpson from day 7 to day 42 (Table 1). Indeed, the k-dominance plot (Figure 2) suggested that a higher diversity evenness was achieved in the sample collected at the later sampling points.

It should be noted that direct comparison of OTUs and taxonomic composition between reported and present study may not be accurate due to differences in approaches and concepts of study. In addition, factors such as environment, treatment, feed additive, antibiotic, age, horizontal gene transfer, hygiene level, diet, type of chicken, geography and climate may also affect the chicken gut microbiota [21,22]. Based on our study, *Firmicutes* was the most predominant phylum found in both ilea and caeca at all ages of chicken (Figure 4). They accounted on average of more than 70% of all bacterial sequences, except in the caeca of 42-day old chicken (48%), an observation consistent with previous reports [10,21,22]. However, these results differ from faecal samples analysed by Singh *et al.* [23] who highlighted that *Proteobacteria* was the most dominant followed by *Firmicutes*, *Bacteroidetes* and *Proteobacteria*.

Comparing ilea and caeca, the former was dominated by bacterial classes such as *Clostridia, Bacteroidia*, unclassified *Gammaproteobacteria* and *Bacilli* while the latter consisted mainly of *Bacteroidia* and *Gammaproteobacteria* (Figure 5) which is in accordance with results reported by Danzeisen *et al.* [22] in caeca. However, Lu *et al*. [13] who studied chicken gut microbiota succession based on partial 16S rRNA sequencing, observed that *Clostridiaceae* was dominant in both ilea and caeca. This contradicts with our finding where *Clostridiaceae* accounted for 5-12% in the ilea during day 7 and 21, and were generally low (<1%) in the caeca throughout the sampling period (Additional file 3).

Similarly, as reflected in the phylum, class and order level, our results showed considerable differences in genera distribution when compared to previous studies. As an example, *Enterobacteria*, *Lactobacilli* and *Enterococci* were found to dominate the small intestines while *Bacteroides*, *Clostridia* and *Lactobacilli* were the main groups of bacteria in the caeca observed from classical reports using culture dependent approaches [6,7,30]. These results also varied when compared with early culture independent methods by Lu *et al.* [13] who found *Lactobacillus* (61.05-86.31%), *Clostridium* (1.11-19.2%) and *Bacteroides* (1.01-2.63%) in ilea. Our findings on the contrary, showed large percentage of *Clostridium* (47-70%) and *Bacteroides* (2-20%) but low percentage of *Lactobacillus* (<4%) (Figure 7). In caeca investigated by Stanley *et al.* [4], a high percentage of *Lactobacillus* (24.38%), *Clostridium* (20.13%) and *Bacteroides* (15.83%) were detected which also consistent in previous reports [3,13,31]. These were comparatively higher to our study (Figure 7) but interestingly, we were able to detect rare genera such as *Alistipes* and *Faecalibacterium* which could not be detected in previous methods. Based on our study, *Alistipes* and *Bacteroides* showed apparent difference in composition as chicken aged. Both are the main bacteria involved in producing short-chain fatty acids (SCFA) [32]. Additionally, *Bacteroides* also plays important role in breaking down complex molecules to simpler compounds which are essential to the growth of host and gut microbiota [33]. In contrast with previous report by Lu *et al.* [13] and Stanley *et al.* [4], *Lactobacillus* which is an important probiotic bacteria in promoting healthy gut were found low throughout all age of the studied sample, thus rendering the need for gut modulation.

In general, a temporal shift in chicken gut commensal occurred within 42 days. Modulation could be best performed during transient phase when the gut microbiota is still unstable and do not have strong core microbiota. However, the fluctuation period from transient to stable community varied between studies. Apajalahti *et al.* [34] reported that the stabilization happened in early stage, as early as day 3, based on bacterial densities in ilea and caeca using flow cytometry method. Lu *et al.* [13] and Gong *et al.* [12] were also in agreement with this statement based on molecular methods. Van der Wielen *et al.* [28] on the other hand, found that fluctuation still happened at low level even after day 11 while Amit-Romach *et al.* [17] suggested that fluctuation of microbiota occurred at day 4 till day 25. Based on the results of the present study, the bacterial richness (Shannon diversity index and inverse Simpson index) (Table 1) was relatively stable between day 14 and 21 in both ilea and caeca. However, a more robust temporal sampling regiment is required to confirm our finding.

In this study, Phylogenetic Investigation of Communities by Reconstruction of Unobserved States (PICRUSt) was used to analyze metagenomes which make predictions based on Greengenes 16S rRNA database and KEGG Orthologs (KO). These data were confirmed with PCA for metagenome (KO) (Figure 8) which also inadvertently mirrored the PCA/PCO from 16S microbiota study reported earlier (Figure 3). Significant difference in sugar and amino acid metabolism pathways and bacterial colonization pathways (e.g. bacterial motility proteins, two-component system and bacterial secretion system) were reported in ilea and caeca, respectively. Utilization of amino sugar and nucleotide sugar is important in chicken metabolism and growth. Amino sugar metabolism specifically is responsible for breaking down protein present in feed to amino acids or di- or tri-peptides [35]. These were then transported from intestinal lumen to epithelial cell for energy. Nucleotide sugar metabolism on the other hand is crucial for purine and pyrimidine synthesis which is vital substrate for deoxyribonucleic acids derivatives (e.g. DNA, RNA). In addition, these components are also needed for producing high-energy nucleotides (e.g. ATP, ADP, AMP) needed for cellular metabolism [36]. Based on Figure 9, we observed that the genes responsible for amino sugar and nucleotide sugar metabolism were up-regulated in ilea compared to caeca (P < 0.05). As reported, ilea are important sites for digestion and nutrient absorption while gut microbiota caeca carried many important roles such as fermentation and breaking undigested substrates [5,27,35]. Miska *et al.* [35] also suggested that in birds, amino acids are mainly absorbed in small intestine which include ilea. Nutrient absorption and active transport which occur mainly in ilea may require substantial amount of energy which can be obtained from ATP derived through nucleotide sugar metabolism.

On the other hand, bacterial motility proteins, two-component system and bacterial secretion system pathways were observed the highest in caeca. Two component systems found commonly in all prokaryotes are equipped with sensor kinase and response regulator to modulate gene expression based on environmental stimulus [37-39]. It is a very complex system which contains many sets of genes responsible for the function. This system responds to various stimuli in the environment including temperature, pH, osmotic level, toxicity and nutrients [38,39]. Interestingly, they are also an important factor for accelerating chicken colonization [37]. Similar to the two component system, higher abundance of bacterial motility proteins and bacterial secretion system were also found in the caeca. Ó Cróinín *et al.* [40] reported that the motility proteins play an important roles in bacterial attachment on epithelial cells and travel to or away from stimulus. Bacterial secretion system, which can be classified into Type I-IV, operates generally on the principal of active transportation of protein from cytoplasm to bacterial surface [41]. They also play crucial roles in gut colonization through invasion on mucosal surface and work closely with flagella assembly and bacterial motility proteins (Figure 9). Intriguingly, both bacterial motility and secretion system are heavily involved in host adhesion, infection and colonization through genes which involved in biosynthesis of fimbriae, flagella, outer membrane, metabolic and lipopolysaccharides [42,43].

In conclusion, the present study showed the development and microbial diversity of ilea and caeca microbiota as the chicken aged. Genes which were related to nutrient absorption, bacterial proliferation and colonization pathways were significantly expressed by the microbiome. The population of beneficial microbes such as *Lactobacillus* was comparatively lower than the potentially pathogenic bacteria such as *Clostridium*, rendering the need of gut modulation to improve the gut health of the chicken.

## Materials and methods

### Chicken and sampling

Hundred and four 1-day-old male commercial Cobb 500 new-born broiler chicks were obtained from reputable farm and supplier. They were inspected upon receive to ensure all chicks were free from any deformity and early signs of disease. Only chicks which were considered healthy were used in the study. Standard Operating Procedure (SOP) of broiler house management was followed throughout the experiment. Cleaning and disinfecting of the chicken cages, feeders, drinkers and feed trough through fumigation were performed before the experiment. In addition, strict hygiene and biosecurity measures were practiced to keep diseases out of poultry. Standard Operating Procedure (SOP) of broiler house management and sampling was followed throughout the experiment based on the Guide for the Care and Use of Agricultural Animals in Agricultural Research and Teaching [44].

Healthy chicks were divided into eight groups with thirteen chicken. Each group was assigned to a cage (3 m x 3 m) that had raised on wire floors and contained a self-feeder and waterer. A 100 W bulb per cage was provided for chicks up to 10 days. Chicken were fed on commercial broiler starter (1 to 21 d) and finisher (22 to 42 d) diets *ad libitum* (Table 2).

**Table 2:** **Composition of the commercial diet fed to broilers**

**Ingredients and composition**	**0 to 21 d**	**22 to 42 d**
Crude protein	21%	19%
Crude fat	3%	3%
Crude fibre	5%	7%
Total ash	8%	8%
Moisture	13%	13%
Phosphorus	0.5%	0.5%
Calcium	0.75%	0.75%

Two chicken were randomly selected from each cage and sacrificed on day 7, 14, 21 and 42. All chicken were fasted sixteen hours before sacrificed. Intestinal contents were removed and pooled for each age to reduce variation between individual. Samples were scrapped aseptically from ileum (2 cm from Merkel’s diverticulum and 2 cm from cecum junction) and cecum (both pairs) by sterile glass slides. All samples were immediately stored in −80°C until further analysis.

### DNA Extraction

Genomic DNA was extracted using the QIAamp DNA Stool Mini Kit (QIAGEN, Germany) by following the manufacturer instructions with some modifications. Initially, mucosal contents were treated with 25 mg/ml of lysozyme (Vivantis, Malaysia) in lysis buffer (20 mM Tris-Cl, pH 8.0; 2 mM EDTA, pH 8.0; 1% Triton X-100) for 30 minutes at 37°C. Samples were then treated with DNase-free RNase (Epicentre, USA). Genomic DNA was extracted in five replicates and the extracts were subsequently pooled. DNA concentration and quality were determined using Quantus Fluorometer (Promega, USA) and gel electrophoresis respectively. They were then stored in −20°C until further analysis.

### 16S rRNA Amplification of V3 region and Illumina Sequencing

The V3 hypervariable region of the 16S rRNA gene was amplified from genomic DNA using forward primer (5′ CCTACGGGAGGCAGCAG 3′) and reverse primer (5′ ATTACCGCGGCTGCTGG 3′) [45]. 6-bp barcode sequence unique to each samples was attached into reverse primer for multiplexing. PCR amplifications were carried out using 50 μl reaction mixtures which contained 25 μl NEBNext High-Fidelity 2X PCR Master Mix (New England Biolabs, USA) (containing 2.0 mM MgCl_2_), 25 μM primer and 50 ng DNA template. The PCR reaction included an initial denaturation step at 98°C for 30 seconds followed by 25 cycles of 98°C for 10 seconds, 62°C for 30 seconds, 72°C for 30 seconds and final extension step at 72°C for 5 minutes in SureCycler 8800 Thermal Cycler (Agilent, USA). The PCR product were analysed on 1.5% agarose gel electrophoresis and bands of the desired size (approximately 330 bp) were purified using a QIAquick gel extraction kit (QIAGEN, Germany). DNA quality and concentration were checked using Quantus Fluorometer (Promega, USA). 16S rRNA gene amplicons were quantified by qPCR using KAPA Library Quantification kit (KAPA Biosystems, South Africa) and Eco Real-Time PCR System (Illumina, USA). The amplicons were normalized, pooled and sequenced on the Illumina Miseq deskstop sequencer (2×151 bp paired-end run) at the Monash University Malaysia Genomics Facility.

### Bioinformatics analysis

Illumina reads were analyzed using mothur software package (v 1.33.3) [46] by following analysis pipeline of Miseq SOP (http://www.mothur.org/wiki/MiSeq_SOP) [47] with some modifications. The first 4-bp on the 5′ end were removed to improve cluster identification in the Miseq [45]. Briefly, read pairs were assembled into contigs. A threshold of *phred* quality score (Q ≥ 25) of the base were chosen for a stringent quality control processing. Any contigs with ambiguous base (N) and longer than 200 bp were culled. Identical or duplicate sequences were merged. Sequences were aligned to SILVA bacteria reference database (SSU_Ref database v.102) [48]. Poorly aligned sequences were removed and overhangs at both ends were trimmed so that they overlap the same region. Unique sequences were screened and further de-noise based on pre-clustered command for up to 2 differences between sequences. Chimera sequences were checked and removed using UCHIME which is pre-loaded in mothur [49]. Sequences were then classified using naïve Bayesian classifier against RDP 16S rRNA gene training set (version 9) with bootstrap cutoff of 80% [50,51]. Sequences classified to unrelated taxon were removed. Operational taxonomic unit (OTU)-based method was used for analysis where sequences were split into bins based on taxonomy and clustered to each bin with cutoff of 0.05 [52]. In order to further reduce noise within the data, rare OTUs which represented by total sequences less than five in all samples were removed. The number of sequences was also normalized to 352,780 for each samples by random subsampling to standardize sampling effort for subsequent alpha and beta diversity analyses. The analysis includes principal coordinate analysis (PCoA) and rarefaction curves. Phyloseq v1.8.2 of R package [53] package in R was used to visualize abundance of bacterial taxonomic composition.

### Ecological and statistical analysis

Alpha diversity and rarefaction curve analyses consists of community diversity (Inverse Simpson and Shannon), richness (OTUs number observed and ACE) were performed using mothur based on summary single command. Beta diversity analysis was performed to investigate the diversity between ilea and caeca. This includes Bray-Curtis distance which was calculated to investigate the relationship between communities′ membership and structure between ilea and caeca at each sampling point. Principal coordinate analysis (PCoA) calculations for this distance were calculated using mothur in order to describe the distances between samples. These coordinates and sample metadata were generated and plotted using PRIMER 6 [54]. K-dominance was also plotted using PRIMER 6 to obtain cumulative percentage in relation to species rank. PCoA analysis was also performed for taxonomic assignment in order to determine the distances between levels of classification.

### Metagenomes prediction

Profiling of predictive gut microbiota was analyzed by using PICRUSt [55]. This was done by first picking OTUs against 13 August 2013 Greengenes database. The biom file was uploaded into the online Galaxy terminal (http://huttenhower.sph.harvard.edu/galaxy/) for pre-processing. The output file was further analysed using Statistical Analysis of Metagenomic Profiles (STAMP) software package [56].

### Nucleotide sequence accession numbers

Sequences of this project have been deposited in the NCBI sequence read archive (http://www.ncbi.nlm.nih.gov/Traces/sra/) under accession numbers Genbank: SAMN03092832-SAMN03092839.

## Additional files

Additional file 1: Table S1. PERMANOVA comparison and P value distribution between ilea and caeca bacterial communities. Additional file 2:
**PCoA of taxonomic classification relationship up to genus level based on Hellinger calculator.**
Additional file 3:
**Bacteria family distributions using V3 amplicon sequencing (n = Top 50 OTUs).** For each timepoint (Day 7, 14, 21 and 42) and part of intestine (I = ilea, C = caeca).
