# Supplementary material for: Deciphering chicken gut microbial dynamics based on high-throughput 16S rRNA metagenomics analyses
Source: Gut Pathog. 2015 Feb 26;7:4. doi: 10.1186/s13099-015-0051-7 (PMC4372169; doi:10.1186/s13099-015-0051-7)
Supplement: Additional file 1: Table S1. — PERMANOVA comparison and P value distribution between ilea and caeca bacterial communities. [file 13099_2015_51_MOESM1_ESM.doc]

**Table S1 - PERMANOVA comparison and P value distribution between ilea and caeca bacterial communities**

All timepoints from ilea and caeca were used as replicates, respectively.

|  | Pseudo-F | P (MC) value |
| --- | --- | --- |
| Jaccard Similarity | 2.9666 | 0.044 |
| Theta YC | 3.9209 | 0.039 |
| Bray-Curtis | 3.3858 | 0.047 |
