# Supplementary material for: Deciphering chicken gut microbial dynamics based on high-throughput 16S rRNA metagenomics analyses
Source: Gut Pathog. 2015 Feb 26;7:4. doi: 10.1186/s13099-015-0051-7 (PMC4372169; doi:10.1186/s13099-015-0051-7)
Supplement: Additional file 2: — PCoA of taxonomic classification relationship up to genus level based on Hellinger calculator. [file 13099_2015_51_MOESM2_ESM.docx]

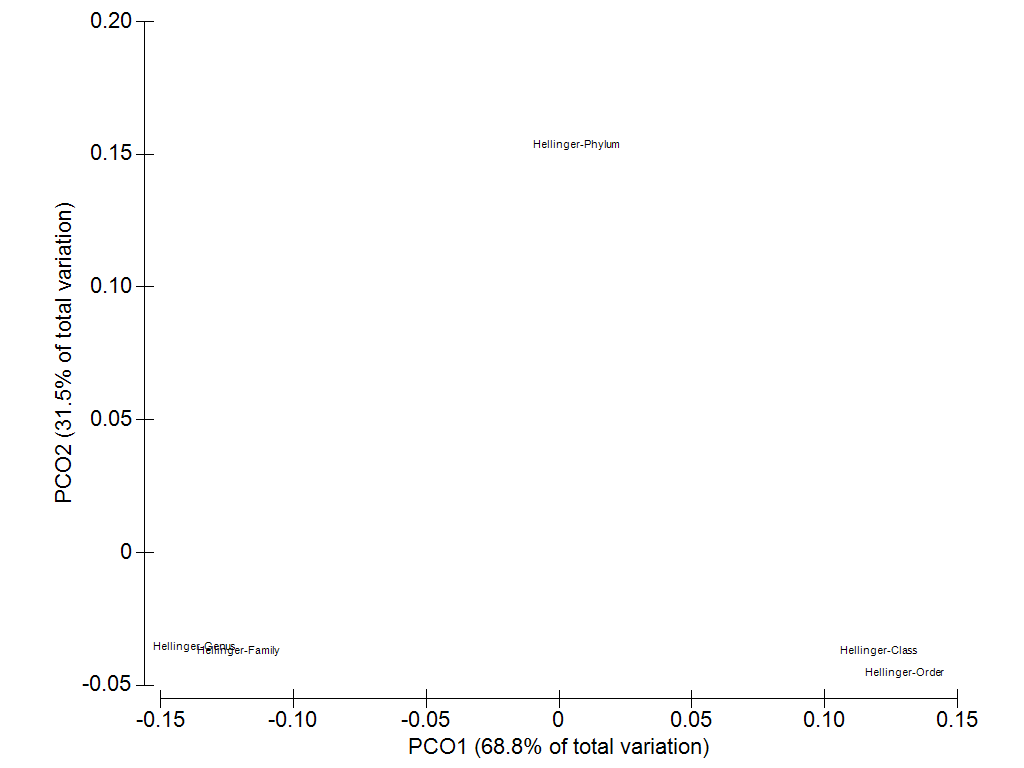


**Additional file 2: PCoA of taxonomic classification relationship up to genus level based on Hellinger calculator.**
