# Supplementary material for: Deciphering chicken gut microbial dynamics based on high-throughput 16S rRNA metagenomics analyses
Source: Gut Pathog. 2015 Feb 26;7:4. doi: 10.1186/s13099-015-0051-7 (PMC4372169; doi:10.1186/s13099-015-0051-7)
Supplement: Additional file 3: — Bacteria family distributions using V3 amplicon sequencing (n = Top 50 OTUs). For each timepoint (Day 7, 14, 21 and 42) and part of intestine (I = ilea, C = caeca). [file 13099_2015_51_MOESM3_ESM.docx]

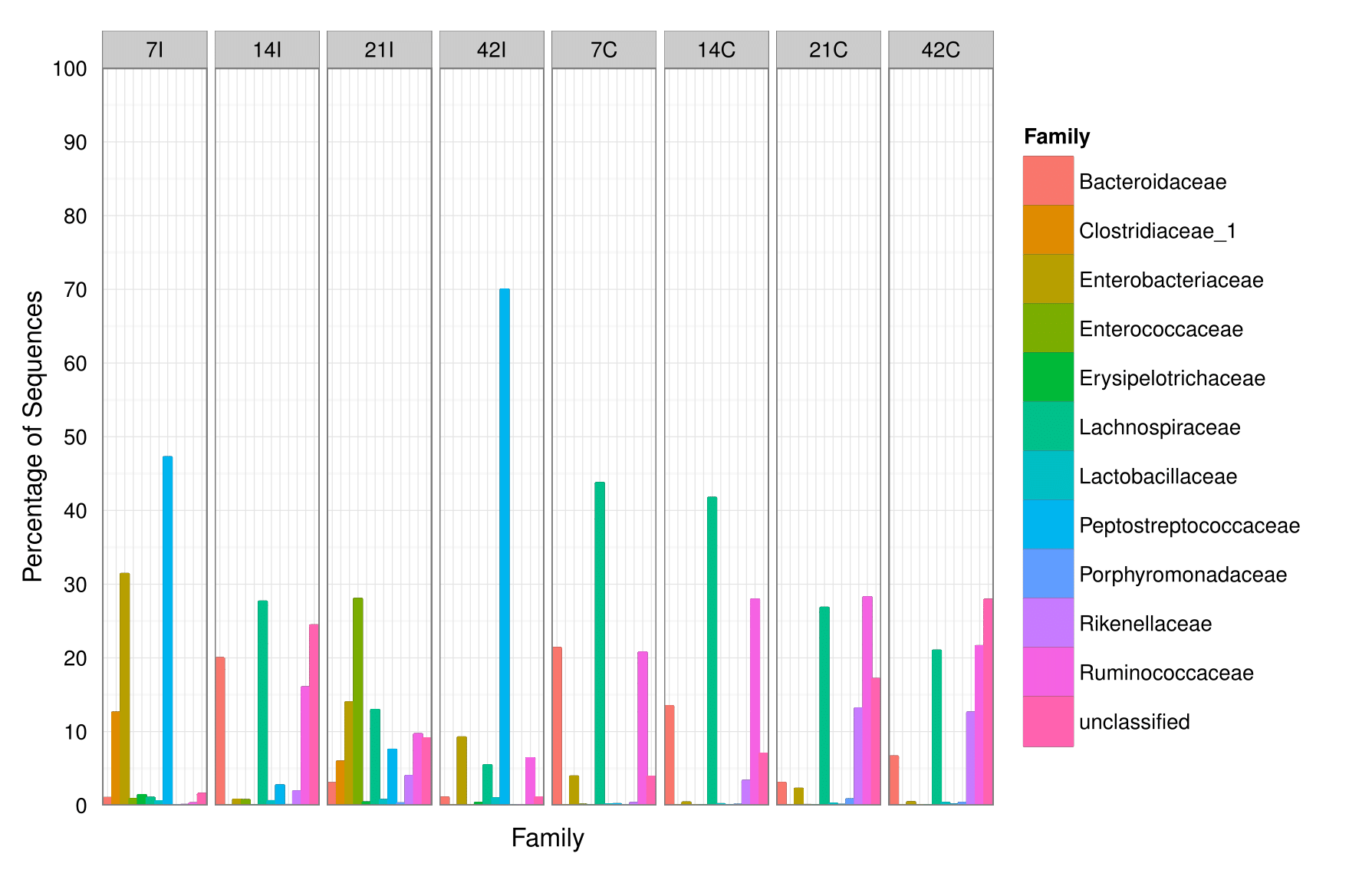


**Additional file 3: Bacteria family distributions using V3 amplicon sequencing (n = Top 50 OTUs).** For each timepoint (Day 7, 14, 21 and 42) and part of intestine (I = ilea, C = caeca).
